# Supplementary material for: Combined nanopore and single-molecule real-time sequencing survey of human betaherpesvirus 5 transcriptome
Source: Sci Rep. 2021 Jul 14;11:14487. doi: 10.1038/s41598-021-93593-y (PMC8280142; doi:10.1038/s41598-021-93593-y)
Supplement: Supplementary file 2 [file 41598_2021_93593_MOESM2_ESM.docx]

**SUPPLEMENTARY FILE 2.)**

**This file contains Supplementary Figures S1-S5.**

**Other supplementary files included:**

**Supplementary File 1.xlsx:** Excel table of **t**he identified transcripts and their characteristics.

**
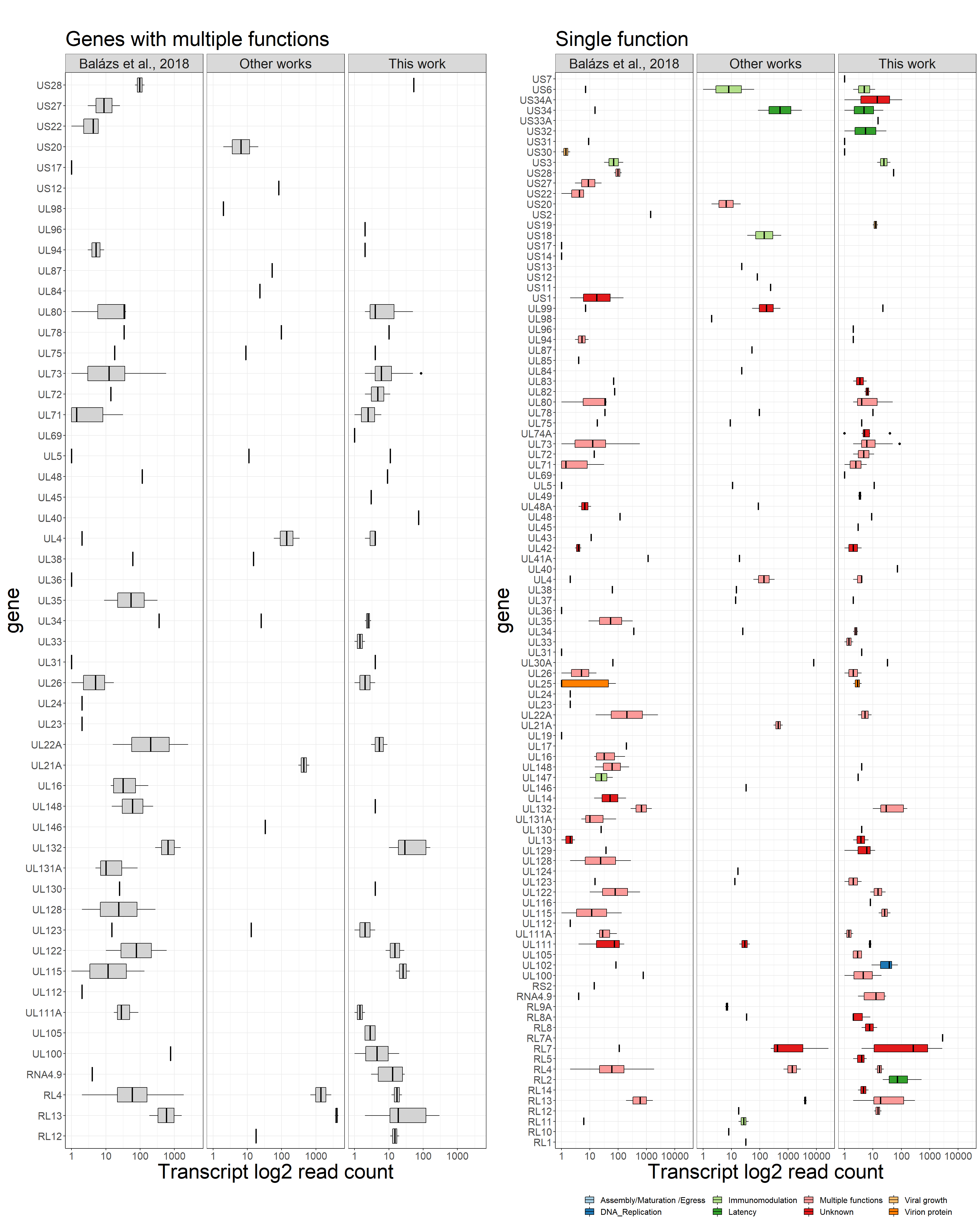
**

**Supplementary Figure S1.** Boxplot of transcript log2 read counts (sum from all samples, x-axis), grouped according to their parent genes (y-axis) and separated according to where the transcript was described in (source). The two panels show whether the genes have multiple or single functions and genes are colored according to gene function as described in a review^1^.


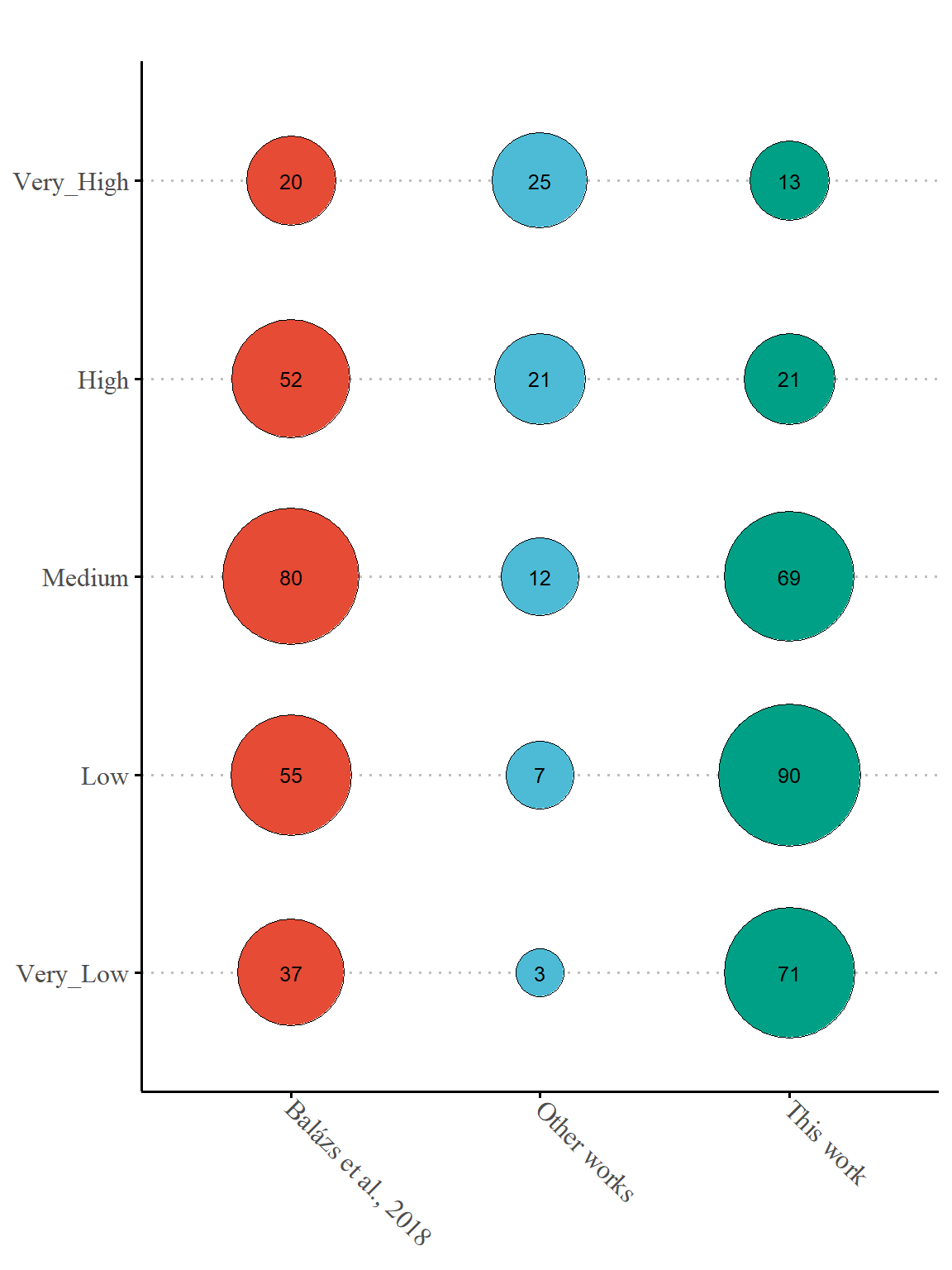


**Supplementary figure S2.** Number of transcripts in each abundance category, according to where the transcript was described in (transcript source).


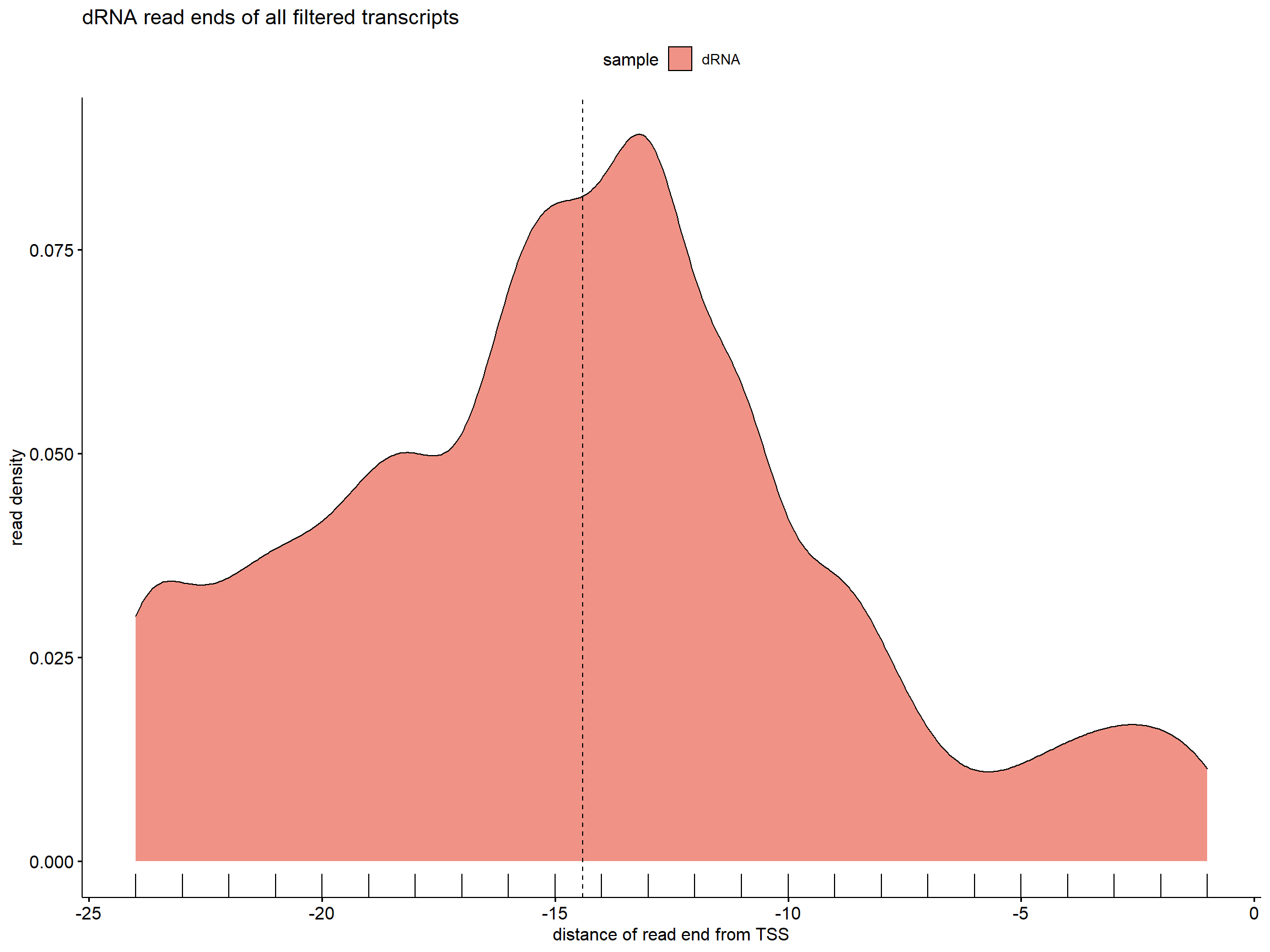


**Supplementary Figure S3.**) Density of the distances of the 5’ ends of the reads from that of the transcript’s (i.e., the zero position indicates the 5’ end of the transcript) in the dRNA sample. Those reads are analyzed only, whose strand could be determined.

**
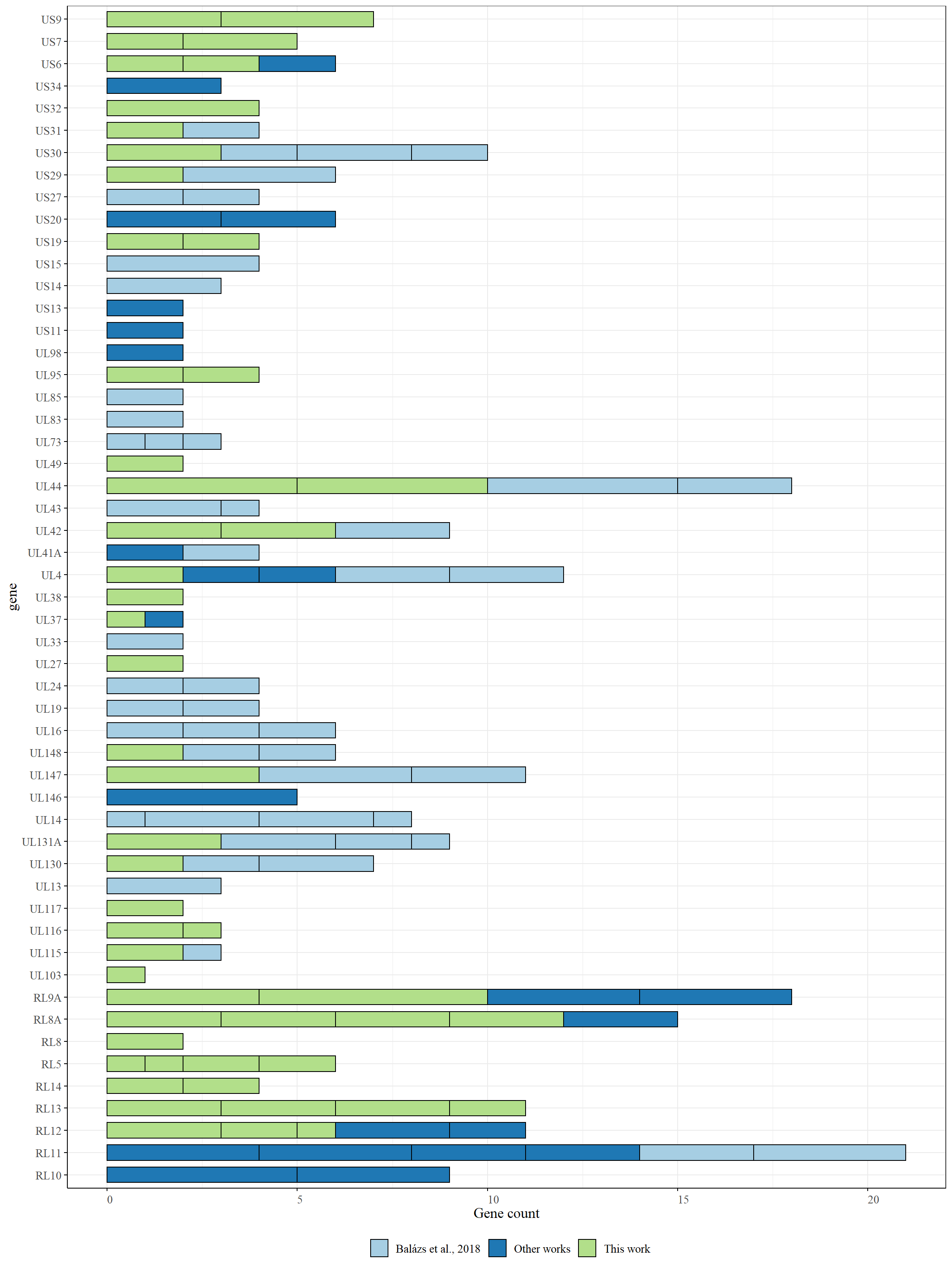
 Supplementary Figure S4.)** Genes that expressed polycistronic transcripts. Each bracket represents a polycistronic transcript, with its first gene shown on the Y-axis. The size of the brackets correspond to the number of genes that the respective transcript carries.

**
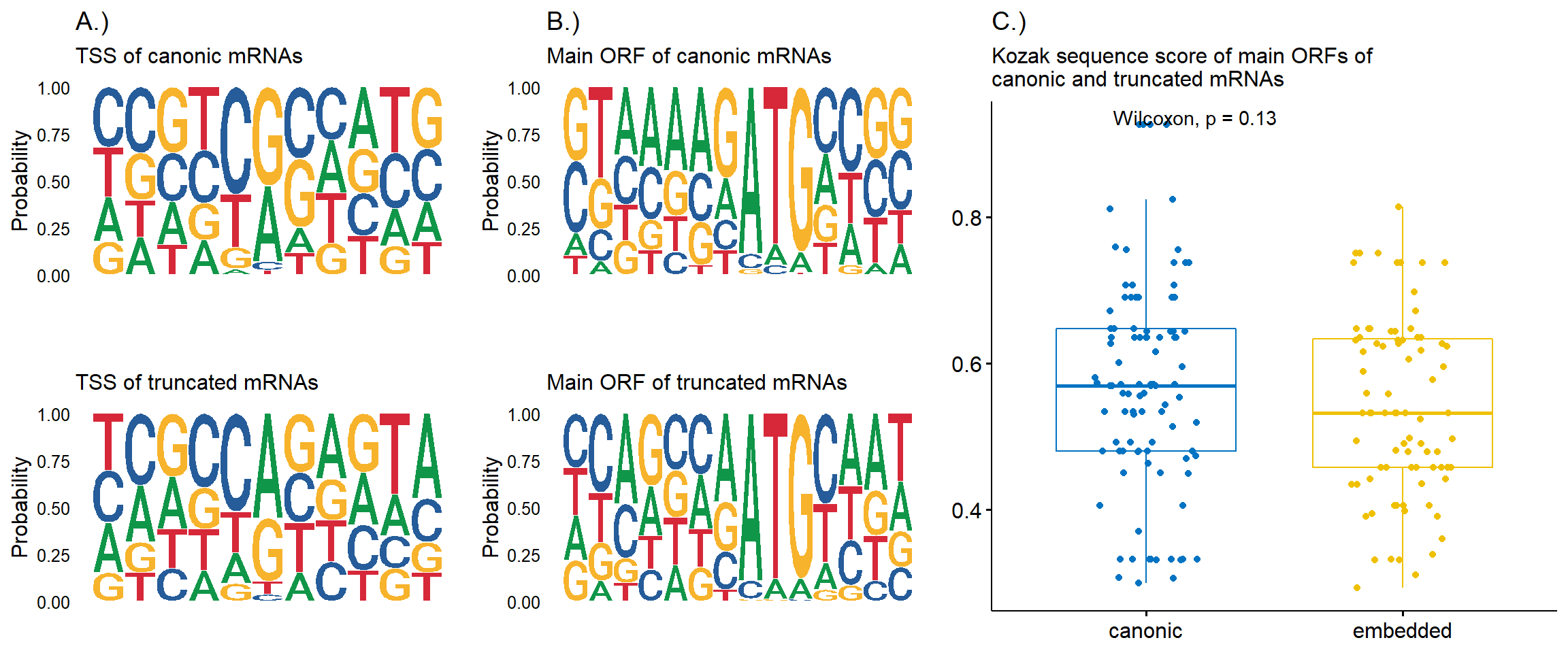
**

**Supplementary Figure S5.** **A.)** Weblogo of the sequences around the TSS of canonic and truncated transcripts (from -5 to +5 from the actual TES); **B.)** Weblogo of the Kozak consensus sequence the main ORFs of truncated and canonic; and **C.)** Kozak sequence score of the main ORFs of truncated and canonic transcripts.

**References**

1. Van Damme, E. & Van Loock, M. Functional annotation of human cytomegalovirus gene products: an update. *Front. Microbiol.* **5**, 218 (2014).
